# Manipulating the temporal locus and content of mind-wandering

**DOI:** 10.1016/j.concog.2020.102885

**Published:** 2020-03

**Authors:** Alice Liefgreen, Marshall A. Dalton, Eleanor A. Maguire

**Affiliations:** a Department of Experimental Psychology, University College London, London, UK; b Brain and Mind Centre, Sydney Medical School, University of Sydney, Sydney, Australia; c Wellcome Centre for Human Neuroimaging, UCL Queen Square Institute of Neurology, University College London, London, UK

**Keywords:** Daydreaming, Illusory motion, Vection, Experience sampling, Mental time travel, Future-thinking, Thought sampling, Scenes, Hippocampus, Episodic memory

## Abstract

The human brain has a tendency to drift into the realm of internally-generated thoughts that are unbound by space and time. The term mind-wandering (MW) is often used describe such thoughts when they are perceptually decoupled. Evidence suggests that exposure to forward and backward illusory motion skews the temporal orientation of MW thoughts to either the future or past respectively. However, little is known about the impact of this manipulation on other features of MW. Here, using a novel experimental paradigm, we first confirmed that our illusory motion method facilitated the generation of MW thoughts congruent with the direction of motion. We then conducted content analyses which revealed that goal orientation and temporal distance were also significantly affected by the direction of illusory motion. We conclude that illusory motion may be an effective means of assaying MW and could help to elucidate this ubiquitous, and likely critical, component of cognition.

## Highlights

• Illusory motion is an effective means of assaying mind-wandering (MW).
• Forward illusory motion facilitates the generation of future-oriented thoughts.
• Backward illusory motion promotes the experience of past-oriented thoughts.
• Illusory motion also affects other phenomenological properties of MW thoughts.

## 1 Introduction

Despite being physically confined to the present, it has been estimated that humans spend up to half of their waking lives in an introspective state decoupled from the external environment, inhabiting an ‘internal world’ which transcends time and space (Killingsworth & Gilbert, 2010). This ubiquitous phenomenon is thought to have a positive influence across areas of cognition including creative thinking and problem solving (Baird et al., 2012). However, it may also be maladaptive, for example, adversely affecting mental health (Andrews-Hanna et al., 2014, Ottaviani and Couyoumdjian, 2013) and educational performance (Smallwood, Fishman, & Schooler, 2007). Despite its pervasiveness and ability to shape cognition, there is still much to learn about this introspective state.

The multitude of terms employed within a range of scientific and philosophical discourse to label this phenomenon, including ‘task-unrelated thought' (Giambra, 1989, Smallwood and Schooler, 2006), ‘stimulus-independent thought’ (Antrobus, 1968, Christoff et al., 2004) and ‘stimulus-independent task-unrelated thought’ (Stawarczyk, Majerus, Maj, Van der Linden, & D’Argembeau, 2011), illustrate how challenging it has been to create a unified account of its properties. One umbrella term that is often used to encompass this type of thinking is mind-wandering (MW), which broadly comprises instances where attention is focused inwards, and the mind meanders between sensory experience and thoughts disengaged from current perception. MW has been found to involve a range of thoughts that vary in terms of content, intentionality, task-relatedness as well as their relationship with external stimuli (Seli et al., 2018). In the present study we characterised as MW those internally-oriented thoughts that may have been, to an extent, related or unrelated to the external environment (Christoff et al., 2016, Smallwood and Schooler, 2006), and which were atemporal, or focused on the past or the future. We included intentional and unintentional thoughts that could have been cued by the context (McVay and Kane, 2010, Seli et al., 2018) but which subsequently evolved into thoughts that were clearly perceptually decoupled. This allowed us to represent MW in arguably naturalistic terms given that real world studies have shown that environmental stimuli regularly trigger MW episodes (Klinger, 2013, Plimpton et al., 2015, McVay and Kane, 2013). Following the conceptualisation proposed by Stawarczyk, Majerus, Maj, et al. (2011), we did not include thoughts that were entirely related or bound to the stimuli or the task at hand and which were, therefore, wholly externally-oriented. This allowed us to distinguish MW from instances of entirely task-related interference (e.g. appraisal of the task) or irrelevant exteroceptive perception (e.g. external distractions). This approach has been successfully employed in previous empirical studies that assessed various features of MW, including, for example, its temporal orientation (Stawarczyk, Cassol, & D’Argembeau, 2013), neural correlates (Stawarczyk et al., 2011, Stawarczyk and D'Argembeau, 2015), ocular correlates (Unsworth & Robison, 2016), association with working memory (Robison & Unsworth, 2015) and cognitive ability (Unsworth & McMillan, 2014).

Due to its extemporaneous and covert nature, the experimental investigation of MW has posed numerous challenges for researchers. A common approach to catching the ‘mind in flight’ involves getting participants to perform simple signal-detection and sustained attention response tasks. Their repetitive nature, low reliance on working memory, and small cognitive load encourages the mind to wander (Smallwood, McSpadden, & Schooler, 2008). These tasks are typically complemented by a variety of experience sampling (ES) methods requiring participants to either recognise and self-report when their mind is ‘off-task’ or be probed by an experimenter regarding the contents of their MW (Smallwood & Schooler, 2015). ES methods can also be ‘retrospective’ and ‘open-ended’, gathering data on people’s MW experiences after task completion and therefore not interrupting the normal flow of MW (Barron et al., 2011, Baird et al., 2011). One issue with retrospective measures is that they rely on participants’ memory and verbal ability to provide accurate accounts of their experiences after they have occurred. However, employing solely self-caught or probe-caught methods may be disruptive to participants’ fluid MW experience (Smallwood & Schooler, 2006). As such, triangulation between different techniques is often deemed the most effective means of gathering data on MW (Christoff et al., 2016, Smallwood and Schooler, 2015).

Considering what we know about MW from this range of measurement techniques, despite being colloquially linked to absent-mindedness, MW has been found to comprise a multiplicity of states with differential content and underlying cognitive architectures (Smallwood and Andrews-Hanna, 2013, Stawarczyk et al., 2011, Wang et al., 2018). Although the phenomenological properties of MW experiences vary markedly between and even within individuals, including its relatedness to the external environment, some common trends have been identified. MW seems to chiefly involve deliberations on current practical concerns and goal-directed planning that is self-relevant (Baird et al., 2011). Moreover, even though the majority of MW experiences comprise realistic events, a smaller percentage of thoughts contain some degree of ‘fantasy’, denoted by implausible elements markedly departing from physical or social reality (Klinger, 2013). Overall, when MW, individuals appear to experience thoughts of a positive valence (Andrews-Hanna et al., 2013, Fox et al., 2013, Smallwood and Schooler, 2015) and of a moderate level of intensity and detail (Andrews-Hanna et al., 2013, Viard et al., 2011). In terms of form, research suggests that people experience an approximately equal number of visual and verbal thoughts (Diaz et al., 2014), and that visual forms of internal mentation are typically from a first-person perspective, thus ‘viewing’ only what lies within one’s visual field (Christian, Miles, Parkinson, & Macrae, 2013).

Perhaps the most consistent MW finding is people’s inclination to engage in mental time travel (MTT; Suddendorf & Corballis, 1997). MTT is the ability to mentally re-experience events that have occurred in the past, and to construct possible future scenarios (Suddendorf et al., 2009, Tulving, 1985). Research investigating the proportion of past- and future-oriented thoughts experienced during MW, however, has produced somewhat conflicting results. While some studies report a ‘prospective bias’ toward future-oriented thinking (Smallwood et al., 2009, Stawarczyk et al., 2013), other studies have observed a high prevalence of past-oriented thoughts during MW (Fransson, 2006, Mason et al., 2007). Despite differences in temporal locus (for a review of this literature see Stawarczyk, 2018), the thoughts experienced during MW have been found to share certain commonalities. For example, both past- and future-oriented thoughts on average appear to involve personally relevant subject matter (Stawarczyk, 2018). Nevertheless, the majority of research suggests that thoughts about the past and the future have somewhat different phenomenological qualities. Thinking about past events, for instance, seems to evoke more visual and sensory details than imagining future events (Addis and Schacter, 2008, D’Argembeau and Van der Linden, 2004), possibly due to their stronger reliance on perceptual information (Johnson, Foley, Suengas, & Raye, 1988). In addition, past-oriented thoughts have been found to have a greater temporal distance from the present than future-oriented MW episodes (Berntsen and Jacobsen, 2008, Stawarczyk et al., 2013). In contrast, future-oriented thoughts appear to be more goal-oriented, thus involving forms of planning, as well as being more realistic (Stawarczyk, Majerus, Maquet, et al., 2011). Compared to past- and future-thinking, atemporal thoughts - defined as concerning subject matter in the here-and-now or with no specific time - have often been overlooked in the MW literature. Research on their prevalence has so far reported mixed results, with some studies suggesting they are as frequent as past- and future-oriented thoughts (Jackson, Weinstein, & Balota, 2013) whereas others have documented their prevalence to be akin to that of past-oriented thoughts, but significantly lower than that of future-oriented thoughts (Stawarczyk et al., 2011, Stawarczyk et al., 2013, Stawarczyk and D'Argembeau, 2016). Even in the latter studies, however, present-oriented thoughts and thoughts with no particular temporal orientation aggregately represented approximately 30% of recorded MW episodes, demonstrating the importance of providing participants with the option of reporting thoughts that are ‘atemporal’ in nature.

Researchers have also explored *how* MW allows us to mentally travel in time to the past and the future. It has been theorised that humans comprehend abstract notions of time through the more concrete domain of space (Casasanto & Boroditsky, 2008). We often ascribe temporal concepts to spatial locations by associating the word ‘past’ to ‘backwards’ and ‘future’ to ‘forwards’ (Casasanto and Boroditsky, 2008, Suddendorf et al., 2009) or by linguistically symbolising temporal concepts in spatial terms (e.g., looking ‘forward’ to the summer). A subjective sense of space might therefore be a crucial component of MTT and could explain how we mentally represent things that we have never experienced, such as future events (Tulving, 2005). Although the precise pattern of space-time mappings partly depends on sociocultural factors, it nevertheless appears to be a universal phenomenon (Boroditsky et al., 2011, Suddendorf et al., 2009).

It is also conceivable that our concept of time may be based, at least in part, on representations of physical experiences in perception and motor action (Casasanto and Boroditsky, 2008, Christoff et al., 2016). This idea is supported by findings in the numerical domain where head as well as eye movements (left to right) during random number generation can predict the magnitude of the number (small to large) produced by participants (Loetscher, Bockisch, Nicholls, & Brugger, 2010). Given this notion, it seems plausible that sensory and motor experiences are coupled with temporal thinking. In line with this idea, recent research demonstrated that visuo-spatial processing and attention impacts the temporal orientation and content of MW. Anelli, Ciaramelli, Arzy, and Frassinetti (2016) revealed that leftward and rightward shifts of spatial attention, induced by prismatic adaptation, facilitated MTT towards past and future respectively. More recently, Vannucci, Pelagatti, Chiorri, and Brugger (2019) illustrated that orienting one’s attention to either the left or right (utilising left-pointing and right-pointing arrows in a vigilance task) steered the temporal focus of self-generated MW towards the past or future respectively. This effect extended to the phenomenological properties of thoughts that were found to differ between temporal orientations; future-related MW episodes were associated with personal current concerns and past events were of greater intensity and more detailed (Vannucci et al., 2019). These findings provide compelling evidence of the flexibility of the temporal focus of MW and its sensitivity to manipulations of spatial attention.

Motivated by the idea that space, motion and temporal constructs may be coupled (Oliveri, Koch, & Caltagirone, 2009), it has been further proposed that thinking about the past and the future could influence people’s bodily movements through space, an idea that offers novel leverage on understanding the cognitive and neural mechanisms involved in MW and MTT (Miles, Nind, & Macrae, 2010). As such, Miles, Nind, et al. (2010) showed that episodes of retrospection induced backward bodily movement and episodes of prospection induced forward bodily movement. This was measured through oscillations in the magnitude and direction of participants’ postural sway when engaging in MW after being instructed to recollect specific memories or construct future events. Similarly, Rinaldi, Locati, Parolin, Bernardi, and Girelli (2016) recorded whole-body movement kinematics and illustrated that people initiated faster steps backward in response to past- than to future-related words and that the converse was true of steps forward. The authors therefore concluded that temporal processing affects physical movements along the sagittal space.

A parallel stream of research explored if the converse was true, namely, whether perceived or physical motion might affect temporal constructs. Aksentijevic, Brandt, Tsakanikos, and Thorpe (2019) provided evidence for this by demonstrating that backward motion-induced mental time travel to the past (induced either through illusory motion, imagined walking or physical walking) improved memory recall for different types of information. Extending this idea to MW, Miles, Karpinska, Lumsden, and Macrae (2010) tested whether forward and backward movement induced future- and past-oriented thoughts respectively. Rather than examining participants’ physical movements in space, the authors embedded a simple signal-detection task within a dynamic visual display that has previously been found to effectively convey illusory self-motion through patterns of optical flow (Aksentijevic et al., 2019, Caruso et al., 2013, Trutoiu et al., 2009). When a large part of the visual field moves, the viewer feels like she is moving whilst the world is standing still (Hettinger, Schmidt-Daly, Jones, & Keshavarz, 2014). In essence, illusory motion is similar to the feeling of sitting on a stationary train and having the impression of moving due to the movement of an adjacent train. Previous studies have found that directionality of illusory motion significantly affected factors such as people’s emotional states and cognition of time (Seno et al., 2012). By exposing participants to these visual stimuli and retrospectively measuring the temporal orientation of participants’ thoughts, Miles, Karpinska, et al. (2010) showed that experiencing backward and forward illusory motion induced self-generated past- and future-oriented thoughts respectively during MW.

Despite the growing number of studies suggesting a link between perceived or actual motion and the temporal locus of MW, the extent to which illusory motion affects other phenomenological properties of MW remains unexplored. Given that spatial attention was found to influence phenomenological properties of thoughts experienced when MW (Vannucci et al., 2019), we expected this to be the case when utilising stimuli inducing illusory physical motion. Therefore, in the present study we first sought to replicate Miles, Karpinska, et al.’s (2010) findings of illusory motion influencing the temporal orientation of spontaneous thoughts in a motion-congruent direction, while also examining atemporal thoughts. We then went beyond previous work to examine the effect of this manipulation on a range of features of spontaneous thoughts, including their temporal orientation, verbal or visual form, perspective, goal orientation, social orientation, whether they were real or fantasy-based, temporal distance, level of detail, and vividness of imagery.

We incorporated aspects of Miles, Karpinska, et al.’s (2010) methodology within a novel paradigm designed to free participants from the demands imposed by performing vigilance tasks and the requirement to self-identify MW thoughts (O’Callaghan et al., 2015, Seno et al., 2011, Smallwood and Schooler, 2015). We believed this novel approach would induce high levels of MW and, combined with a triangulation of ES methods (namely probe-caught, retrospective, and open-ended), would enable us to collect comprehensive information about MW experiences. In addition, by retaining elements of Miles, Karpinska, et al.’s (2010) methodology, we ensured that our results and conclusions would be comparable.

Based on the findings of Miles, Karpinska, et al. (2010) we predicted that directional vection would facilitate direction-congruent MTT. More specifically, exposure to forward illusory motion would facilitate future-oriented thoughts and backward illusory motion would promote past-oriented thoughts. Our predictions about the effect of illusory motion on the diverse phenomenological features of thoughts were made based on pre-existing literature on MW. We hypothesised that future-oriented thoughts associated with forward illusory motion would be of a goal-oriented nature (Andrews-Hanna et al., 2013, D’Argembeau and Van der Linden, 2004, Stawarczyk et al., 2013). Backward illusory motion was expected to induce past-oriented thoughts, containing greater levels of detail and imagery (Andrews-Hanna et al., 2013, Fox et al., 2013, Smallwood et al., 2016). We also predicted that, regardless of illusory motion, thoughts would be experienced from a first-person perspective and comprise positive, realistic and meaningful self-oriented content rather than being socially-oriented and of fantastical nature (Christoff et al., 2016, Corballis, 2013, Smallwood and Schooler, 2015).

## 2 Materials and methods

### 2.1 Participants

In order for our results to be comparable to those of Miles, Karpinska, et al. (2010), our sample size mirrored that employed in their study. Thirty-nine healthy participants (20 females; *M* age = 27.1, SD = 9) were recruited through a University College London volunteer database. Participants were randomly allocated to one of three groups that each comprised thirteen participants. Employing a between-groups design, each participant group was exposed to a different visual stimulus. All participants gave informed written consent prior to participation, in accordance with the approval of the local research ethics committee.

### 2.2 Stimuli

**Fig. 1:**
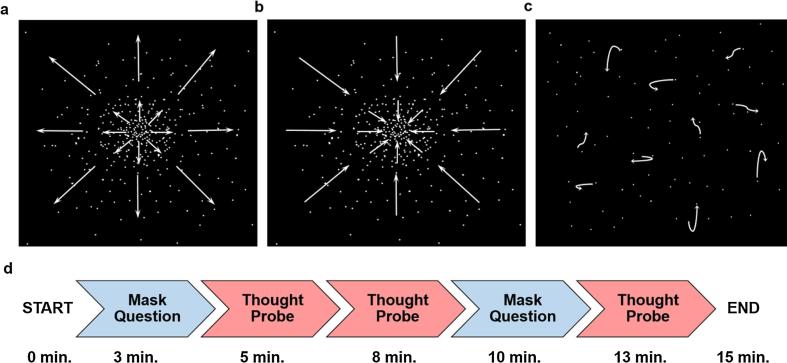
Stimuli and thought sampling paradigm. During the experiment participants viewed an animated star field that induced either (a) the feeling of forward motion, (b) the feeling of backward motion or (c) no perceivable sense of motion. (d) The thought sampling procedure required participants to provide one-sentence answers either to simple trivia ‘mask’ questions or thought probe questions which required the participant to disclose what was on his/her mind at the time of asking.

Three distinct visual stimuli (one for each participant group) were produced using Cogent 2000 (Wellcome Centre for Human Neuroimaging and Institute of Cognitive Neuroscience, UCL, London, UK), operated within MATLAB R2014a (MathWorks Inc., USA). Stimuli were animated star fields created by the movement of 1000 randomly allocated white dots (size = 1 pixel each), moving at 25 frames per second on a black background (Fig. 1). The speed of the dots varied slightly as they moved in steps from the centre to the periphery of the screen. Each step occurred in sync with the monitor frame rate (60 Hz). In one condition, the dots moved on a linear trajectory toward the centre of the screen (centripetally, conveying a sense of backward motion). In the second condition, the dots moved on a linear trajectory away from the centre of the screen (centrifugally, conveying a sense of forward motion). The angle of motion of the stars was equal to their angular position about the centre, meaning they moved directly to/from the centre point. We also included a third, no-vection, condition where the dots were designed to move in a randomised and incoherent manner around the screen to eliminate any perception of intelligible or directional motion. We reasoned that the lack of directional illusory motion would affect the experience of MTT which in turn would make it less likely that MW would occur. On this basis, we predicted fewer MW thoughts during the no-vection condition compared to the forward and backward vection conditions, and hence more stimulus-dependent thoughts.

Although participants were instructed to keep their eyes on the centre of the screen to optimally experience the illusory motion, no vigilance task or fixation point was embedded within any of the stimuli in order to maintain the naturalistic flow of the star field and to facilitate MW. The stimuli were initially piloted (N = 4) to ensure that they induced the desired direction of motion in each condition.

### 2.3 Procedure

Participants were informed they were taking part in an experiment aimed at investigating the effect of dynamic environments on attention and cognition. The primary interest in MW was initially withheld in order not to hinder the spontaneous nature of this phenomenon. Participants were further told they would be asked simple questions during a 15-minute visual stimulus presentation and they should provide, for each question, a brief one-sentence answer aloud and without taking their eyes off the centre of the screen. There would be “general” questions requiring them to briefly report what was on their mind (thought probe). They were told these were simply control questions, but should nevertheless be answered honestly and with a short one-sentence description. In addition, they were informed that they would be asked more specific questions and were provided with an example. These questions were designed to be simple and answerable in one short sentence without requiring extensive thinking, so as not to disrupt any MW. By ‘masking’ the thought probe questions with more specific trivia questions, the idea was that participants would be less self-aware of their own thoughts, thus maximising the spontaneity of MW. In order to check that participants understood the procedure, they were asked two practice questions prior to commencing the experiment.

Participants were then asked to sit in a chair placed 200 cm from a large screen (145 cm × 110 cm) onto which the stimulus was displayed, so that the centre of the star field was approximately at eye level. These measurements were employed as they were previously utilised by Miles, Karpinska, et al. (2010) to effectively induce illusory motion. All windows in the room were obscured and lights were switched off to create total darkness and facilitate visualisation of the display. The stimulus was projected utilising an EPSON EH projector with 3LCD Technology and RGB liquid crystalliser for optimal image quality. The projector was placed to the right of, and just behind, the participant, and was thus outside their field of view. The researcher placed two audio-recorders by the participant’s chair, started the experimental stimulus, and sat in a position that allowed them to monitor the participant’s attentiveness without creating any distraction. The researcher then proceeded to ask the questions at designated intervals (Fig. 1). Participants were asked ‘what were you thinking about now, just before I asked?’ approximately 5, 8 and 13 min from stimulus onset and answers were noted by the researcher. They were also asked two trivia questions (‘can you count to ten for me as fast as you can?’ and ‘what are the main ingredients in a cup of coffee?’) approximately 3 and 10 min from stimulus onset. The timing between questions was chosen following feedback from the pilot study, wherein participants reported it to be a sufficient period for spontaneous thoughts to arise.

Each participant group saw one stimulus type – the backward motion, the forward motion or the no-vection motion. Following the 15-minute stimulus presentation, the experimenter proceeded to gather more detailed information on the three sampled thoughts through further ES methods. First, the one-sentence responses to the three thought probes were read aloud to participants one at a time. Once corroborated, they were asked to describe the thought out loud in as much detail as possible and were occasionally probed in a general manner as encouragement. Subsequently, participants were verbally administered a questionnaire in order to gather more detailed information on the content of the thought. Participants were instructed to answer each question according to how they remembered experiencing the thought during the stimulus presentation, and without subsequent elaboration. The questionnaire was delivered in a standardised way to all participants with questions and options being read out loud, and answers recorded on a hard copy of the questionnaire. This questionnaire allowed the researcher to measure the dependent variables of interest through the use of Likert scales representing thought content ratings as well as through multiple choice and open-ended questions. For example, participants were asked to report the temporal orientation of their thought by classifying it according to whether it was future-oriented (took place in a future moment following the experiment), past-oriented (took place in a past moment classified as preceding the experiment) or atemporal (with no temporal orientation). Following the verbal questionnaire, participants were debriefed, and all reported that during the task they had no knowledge of the researcher’s primary interest in MW and that they had not subsequently elaborated on the thoughts compared to when they first experienced them during the stimulus presentation.

After the experimental session, the thought descriptions were transcribed verbatim and coded by a rater (who was blind to which condition the participant was in) for categorical features that were less suitable for the participants to rate themselves, specifically: whether thoughts were stimulus-dependent, task-dependant, stimulus-related, or stimulus/task-independent (measuring the extent to which thoughts were internally- or externally-oriented) were/were not goal-oriented (whether the subject matter of the thought involved reaching a particular task-unrelated goal or involved devising a plan to reach a goal, both short-term and long-term), were socially-oriented or self-oriented (whether the thought involved or concerned other people or social interactions with someone other than solely the participant him/herself), and whether they were realistic and part of our social and physical reality, or of a fantastical nature (realness). For a list of all the thought features of interest and their measurement see Table 1.

**Table 1:** Thought features and their measurement.

	Feature	Sampling Method	Measurement
***Categorical Choices***	*Stimulus/Task Dependency	Thought subject matter was coded from transcription for stimulus/task dependency	Quaternary classification (stimulus-dependent, task-dependant, stimulus-related, stimulus/task-independent)
	**Temporal Orientation**	Participant was asked “When did the subject matter of your thought take place”	Ternary choice (future, past, neither/present)
	**Modality**	Participant was asked “Was the thought in the form of visual images or words, or a mixture of both?”	Ternary choice (visual, verbal, both)
	**Verbal Modality**	Participant was asked “If verbal, was it experienced as whole sentences or random words?”	Ternary choice (sentences, words, both)
	**Visual Modality I**	Participant was asked “If visual, was it in the form of a scene or a single object?”	Ternary choice (scene, object, both)
	**Visual Modality II**	Participant was asked “If visual, was it moving or static?”	Ternary choice (moving, static, both)
	**Visual Modality III**	Participant was asked “If visual, was the thought experienced from a first-person perspective, so from your own perspective, or from a third-person perspective, so as if you were an observer?”	Binary choice (first-person, third-person)
	*Goal Orientation	Thought subject matter was coded from transcription for goal orientation	Binary classification (goal-oriented, non goal-oriented)
	*Social Orientation	Thought subject matter was coded from transcription for social orientation	Binary classification (socially-oriented, self-oriented)
	*Realness	Thought subject matter was coded from transcription for realness	Binary classification (real, fantasy)
***Likert Ratings***	**Temporal Distance**	Participant was asked “If it took place in the past/future, was it…”	Likert scale (0 = earlier today/later today to 5 = more than 3 years ago/more than 3 years ahead)
	**Emotional Valence**	Participant was asked “The emotions relating to this thought were…”	Likert scale (0 = extremely negative to 5 = extremely positive)
	**Emotional Intensity**	Participant was asked “The intensity of the emotions relating to this thought were…”	Likert scale (0 = not intense at all to 5 = very intense)
	**Detail**	Participant was asked “In terms of content and coherence, I would characterise this thought as highly detailed”	Likert scale (0 = strongly disagree to 5 = strongly agree)
	**Vividness of Imagery**	Participant was asked “The mental imagery relating to this thought was…”	Likert scale (0 = no imagery to 5 = perfectly clear and vivid)

* These thoughts were categorised by the experimenter and second-coded by an independent rater (for inter-rater agreement see the end of Section 2.5). The remaining features were categorised/rated by the participants.

### 2.4 Scoring

In order to accommodate a range of data types, we used two scoring methods. For the categorical choices [i.e., stimulus/task dependency (stimulus-dependent, task-dependent, stimulus-related, stimulus/task-independent), temporal orientation (future/past/atemporal), modality (verbal/visual/both), verbal modality (words/sentences/both), visual modality I (scene/object;), visual modality II (moving/static), visual modality III (first or third person perspective) goal orientation, social orientation, realness] the number of thoughts each participant experienced (out of three thoughts) for that feature were counted (e.g. one participant experienced 1 goal-oriented thought and 2 non goal-oriented thoughts). This allowed us to subsequently compute the proportion of thoughts pertaining to each feature for each condition and carry out meaningful between-condition comparisons. Each participant’s ratings of temporal distance, emotional valence, emotional intensity, detail, and vividness of imagery were averaged across the three MW thoughts in order to obtain a mean score on each of these variables for each participant. Subsequently, the mean score for each variable was computed for each condition to allow for between-condition comparisons.

### 2.5 Identification of MW thoughts

Following the conceptualisation of Stawarczyk, Majerus, Maj, et al. (2011), we characterised thoughts along the dimensions of “task relatedness” and “stimulus dependency”. As such, all 117 thoughts were categorised as being either stimulus-dependent, task-dependant, stimulus-related or stimulus/task-independent. These were mutually exhaustive and exclusive categories. A thought was categorised as being stimulus-dependent if it comprised content that was entirely perceptually coupled to the stimuli the participant was being exposed to, for example P.23 described thinking “…*that the dots look like flies and then at the end they started to look like snowflakes falling and then they looked like flies again. I was just wondering if they were meant to be like flies or not*”. Task-dependent thoughts comprised content that was entirely related to the task (e.g. the filler questions). For example, P. 36 reported thinking “…*that when you asked me before about the ingredients in coffee and I just replied coffee that it actually can’t happen and that you probably need water as well*”.

Thoughts were classified as stimulus-related if they stemmed from the stimuli but developed into a thought of original content that was perceptually decoupled. For example, P. 38 described “…*thinking about lying in bed and I was probably falling asleep on her chest trying to watch the Night Manager. I started thinking about the Night Manager because the dots on the screen made me think of the scene where he is smoking a cigarette outside in the series and it is all starry and nice in the sky. I was thinking about how warm and comfortable I felt in bed like that*”. Finally, a thought was classified as being stimulus/task-independent if the subject matter had no relation to the stimuli or task and was therefore perceptually decoupled from the outset. An example could be an autobiographical memory, such as that recalled by P.32 regarding “…*going to dinner with my friend and we were sitting on our couch and I could see the couch and the stool in front of us where we had plates with leftover food on them and I could see cartons of cigarettes that were a bit discarded and the window was open so it was basically the scene from last night but I could see the objects on the table and everything.”*

Partitioning thoughts into these four categories allowed us to identify the proportion of thoughts that could be characterised as MW utilising our definition as outlined in Section 1. These were the thoughts labelled as either stimulus-related or stimulus/task-independent, as their content was either entirely perceptually decoupled or, despite being initially triggered by the stimuli, evolved into a perceptually decoupled thought that was internally- rather than externally-oriented. Given that the focus of the present study was to explore the effect of vection on the phenomenological properties of thoughts experienced when MW, those thoughts that were labelled as either stimulus-dependent or task-dependent were excluded from the analyses due to their sole focus on the external environment (Stawarczyk, Majerus, Maj, et al., 2011).

To assess the validity of our categorisation method, 60.6% of thoughts (71 out of 117) were randomly selected and second-coded on all experimenter-rated thought features (stimulus dependence, task dependence, stimulus relatedness, stimulus/task independence, goal orientation, social/self-orientation and realness) by an independent rater blind to the purpose of the experiment and the conditions. Cohen’s kappa coefficient was computed for each of these features, and showed that there was high inter-rater agreement on all of the variables: stimulus dependence, κ = 0.94, *p* < 0.0001; task dependence, κ = 1, *p* = 0.0; stimulus relatedness, κ = 0.95, *p* < 0.0001; stimulus/task independence, κ = 1, *p* = 0.0; goal orientation, κ = 0.97, *p* < 0.0001; social/self orientation, κ = 0.9, *p* < 0.0001 and realness, κ = 0.93, *p* < 0.0001.

### 2.6 Data analyses

Data were analysed using RStudio version 1.145 (RStudio Team, 2015). Chi-Square Tests of Independence were used to detect between-condition differences in the proportion of thoughts relating to each categorical feature (Table 1; stimulus/task dependency, temporal orientation, modality, goal orientation, social orientation and realness), with descriptive analysis being reported for sub-categories of these features (Table 1; verbal modality, visual modality I and II and perspective). We used One-Way ANOVAs to examine between-condition differences in thought features measured on a continuous Likert-scale (Table 1; temporal distance, emotional valence, emotional intensity, detail and vividness of imagery).

## 3 Results

### 3.1 MW thoughts

We found that the proportion of people who experienced MW (comprising thoughts labelled as either stimulus-related or stimulus-independent), differed significantly between the three conditions, χ^2^ (2) = 19.07, *p* < 0.0001. Post-hoc Fischer’s exact tests with adjusted alpha values corrected for multiple comparisons illustrated the significant difference lay in the proportions of MW experienced in the forward vection and no-vection condition, *p* = 0.004 and between those experienced in the backward vection and no-vection conditions, *p* = 0.0002. As such, and as is evident on Table 2, participants in the no-vection condition experienced significantly more externally-oriented (stimulus-dependent and task-dependent), and fewer MW, thoughts than participants in the other two conditions. This finding accords with our prediction that the no-vection condition would not induce much MW. This no-vection result also means that, in terms of MW thoughts, this condition is under-powered relative the forward and backward vection conditions. As such, our analyses hereafter focus on the two main conditions of interest, examining how forward and backward vection affected the phenomenological properties of MW.

**Table 2:** Number of thoughts categorised as stimulus-dependent, task-dependent, stimulus-related and stimulus/task-independent within each condition.

Vection Condition	Stimulus- Dependent	Task-Dependent	Stimulus-Related	Stimulus/Task- Independent	**Total MW Thoughts**
Forward	7	0	7	25	**32**
Backward	4	0	18	17	**35**
No-vection	18	2	2	17	**19**

*Note:* total MW thoughts is the sum of stimulus-related and stimulus/task independent thoughts.

### 3.2 Temporal orientation

**Fig. 2:**
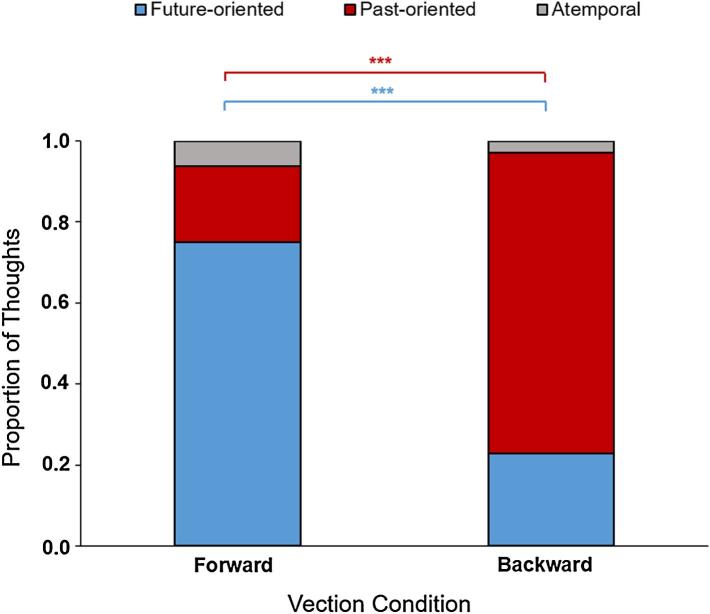
Temporal orientation of MW thoughts. The proportion of future- (blue), past-oriented (red) and atemporal (grey) thoughts experienced by participants exposed to either forward or backward vection. ***p < 0.001. (For interpretation of the references to color in this figure legend, the reader is referred to the web version of this article.)

We first examined whether illusory motion had the predicted effect on the temporal orientation of MW thoughts. There was a significant difference in the proportion of future-oriented thoughts between conditions, χ^2^ (1) = 16.19, *p* < 0.0001, and in the proportion of past-oriented thoughts between conditions, χ^2^ (1) = 18.5, *p* < 0.0001. There was no significant difference in the proportion of atemporal spontaneous thoughts experienced by participants in the two conditions, χ^2^ (1) = 0.006, *p* = 0.93. As can be seen from Fig. 2, participants in the backward vection condition experienced a greater proportion of past-oriented thoughts (0.74) than participants in the forward vection condition (0.19), who in turn experienced a greater proportion of future-oriented thoughts (0.75) than their counterparts in the backward vection condition (0.23). Participants in both conditions experienced a very small proportion of atemporal thoughts (forward vection condition, 0.06; backward vection condition, 0.03).

In summary, our findings replicated those of Miles, Karpinska, et al.’s (2010) whereby exposure to forward illusory motion led to participants experiencing a greater number of future-oriented thoughts and, conversely, exposure to backward illusory motion facilitated the experience of past-oriented thoughts when MW.

We next examined whether the direction of illusory motion affected other phenomenological features of thoughts experienced when MW.

### 3.3 Modality

No significant between-condition difference was apparent in the proportion of thoughts experienced by participants in a visual-only modality (forward vection 0.4; backward vection 0.4), in a verbal-only modality (forward vection 0.1; backward vection 0.06), or as a mixture of visual and verbal modalities (forward vection 0.47; backward vection 0.54), χ^2^ (1) = 1.04, *p* = 0.59.

#### 3.3.1 Modality descriptives

Across conditions, of the thoughts with verbal elements, 70% were in the form of sentences, 20% in the form of words and 10% as a mixture of both. Of the thoughts with visual elements, 72% were experienced as a scene, 16% in the form of single objects and 11% as a mixture of both. Moreover, 49% were experienced as moving, 41% as static and 10% as a mixture of both, while 90% were experienced from a first-person perspective and the remaining 10% from a third-person perspective.

### 3.4 Goal orientation

**Fig. 3:**
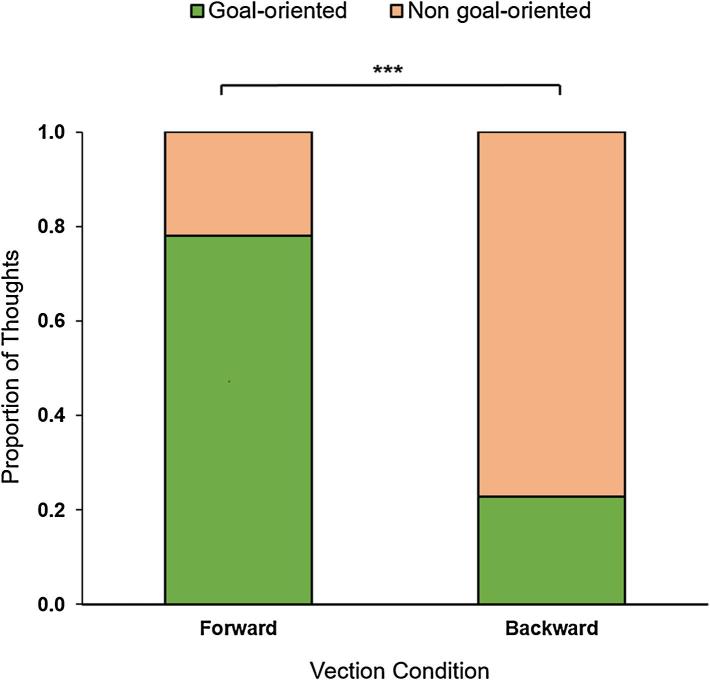
Goal orientation of MW thoughts. The proportion of goal-oriented (green) and non goal-oriented (orange) thoughts experienced by participants exposed to either forward or backward vection. ***p < 0.001. (For interpretation of the references to color in this figure legend, the reader is referred to the web version of this article.)

We found a significant difference in the proportion of goal-oriented thoughts experienced by participants exposed to forward vection (0.78) and participants exposed to backward vection (0.23), χ^2^ (1) = 18.28, *p* < 0.0001 (Fig. 3).

### 3.5 Social orientation, realness

We found no significant difference in the proportion of thoughts concerning ‘self-oriented’ subject matter between the forward (0.78) and backward-vection (0.66) conditions, χ^2^ (1) = 0.73, *p* = 0.39. Similarly, there was no significant difference in the proportion of thoughts of ‘real’ subject matter experienced by participants exposed to forward vection (0.94) and backward vection (0.89), χ^2^ (1) = 0.1, *p* = 0.75.

### 3.6 Temporal distance, emotional valence, emotional intensity, level of detail, vividness of imagery

**Fig. 4:**
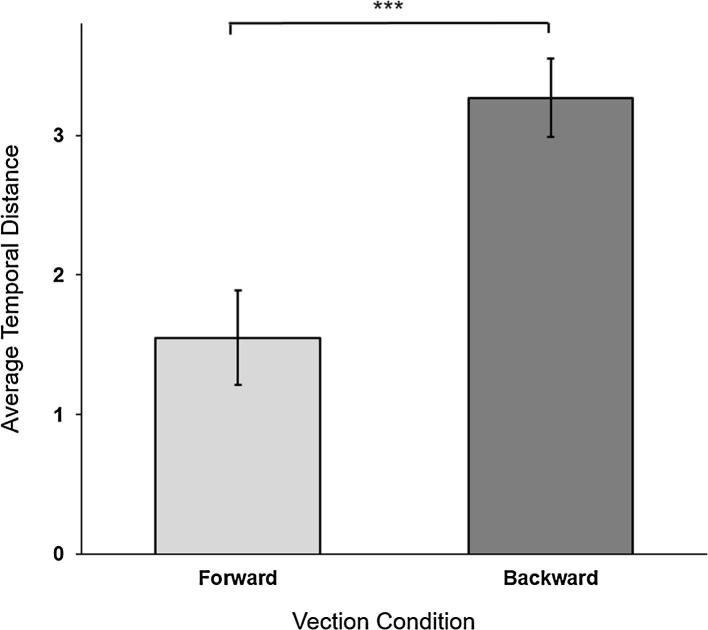
Temporal distance of MW thoughts. The average temporal distance rating of MW thoughts associated with forward (light grey) and backward (dark grey) vection. The rating options were 0 = earlier today/later today up to 5 = more than 3 years ago/more than 3 years ahead. Error bars are SEM. ***p < 0.001.

A significant difference was found in the mean temporal distance of thoughts experienced by participants in the forward vection condition (*M* = 1.55, SD = 1) compared to those experienced in the backward vection condition (*M* = 3.3, SD = 1.2), *F* (1, 23.3) = 15.4, *p* = 0.0007 (Fig. 4). Backward vection induced thoughts that extended further in time than the thoughts elicited during forward vection.

No significant difference was evident in the mean emotional valence ratings of MW thoughts during forward vection (*M* = 3.2, SD = 0.7) and backward vection (*M* = 3.3, SD = 0.5), *F* (1, 22.8) = 0.07, *p* = 0.79. Similarly, there was no difference in the mean emotional intensity ratings for the two conditions (forward *M* = 2.04, SD = 1) (backward *M* = 2.05, SD = 1), *F* (1, 23.8) = 0.001, *p* = 0.98. Finally, mean ratings of level of detail (forward *M* = 3.3, SD = 0.7; backward *M* = 3.2, SD = 0.7), *F* (1, 23.8) = 0.23, *p* = 0.64, and mean ratings of vividness of imagery (forward *M* = 2.7, SD = 1.2; backward *M* = 3.4, SD = 1.3), *F* (1, 23.9) = 2.04, *p* = 0.17 did not differ significantly between the two vection conditions.

The above findings contrast with previous characterisations of past-oriented thoughts as being richer in detail than future-oriented thoughts (D’Argembeau and Van der Linden, 2004, D’Argembeau and Van der Linden, 2006). One could argue that this lack of difference between the two vection conditions in terms of the level of detail of thoughts could be due to past-oriented thoughts (which were more prevalent in the backward vection condition) being significantly more temporally distant than future-oriented thoughts (which were more prevalent in the forward vection condition). Temporally distant thoughts in both the past and future have been posited to contain fewer contextual and sensorial details than temporally close events (Johnson et al., 1988, Trope and Liberman, 2003, Christian et al., 2013). To explore this possibility, we ran a univariate general linear model with one fixed factor (vection condition), one covariate (temporal distance) and one dependent variable (level of detail). We found no significant effect of either temporal distance *F* (1, 62) = 2.45, *p* = 0.12, or condition *F* (1, 62) = 2.45, *p* = 0.52, on level of detail of thoughts. Perhaps of more relevance for explaining this null finding is research showing that what increases the level of detail of thoughts is not temporal distance per se but familiarity with the places involved in the thoughts (Arnold et al., 2011, D’Argembeau and Van der Linden, 2012). Given that past-oriented thoughts are typically composed of memories, and a significant amount of the future-oriented thoughts in our sample involved planning or were of goal-oriented in nature, backward and forward-oriented thoughts likely encompassed familiar locations. This could potentially explain the observed lack of difference in level of detail between the vection conditions.

## 4 Discussion

To the best of our knowledge, this is the first study to examine how the direction of illusory motion affects the phenomenological properties of thoughts experienced during MW. We did this by employing a novel thought sampling paradigm designed to facilitate MW by unconstraining participants’ exogenous attention as much as possible. This task was effective in generating thoughts classifiable as MW within our definition, as participants exposed to either forward or backward vection experienced MW in more than 80% of the thought probe trials. These results provide encouraging support for the use of illusory motion as a tool to capture the mind in flight, given that previous studies utilising vigilance tasks reported a percentage of mind-wandering occurrence closer to 20% (e.g. Stawarczyk, Majerus, Maquet, et al., 2011). In addition, we replicated the findings of Miles, Karpinska, et al. (2010), by showing that forward vection induced thoughts about the future and backward vection elicited thoughts about the past. Our results also move beyond those of Miles, Karpinska, et al. (2010) in several ways. First, we found that no-vection stimuli provoked significantly fewer MW thoughts, aligning with our prediction that this condition would not induce much MW. This result illustrates how a relatively small change in the movement of visual stimuli can significantly curtail the occurrence of MW. Second, we characterised the MW thoughts induced by either forward or backward vection, finding differences and also commonalities in thought features which we now discuss in turn.

Participants exposed to forward illusory motion experienced more goal-oriented thoughts than those exposed to backward vection, in line with previous research showing that future-oriented thoughts are more strongly related to goal-pursuit and planning than other MW episodes (Andrews-Hanna et al., 2013, Baird et al., 2011, Stawarczyk et al., 2013). In contrast, the content of past-oriented thoughts typically relates to reliving autobiographical memories rather than goal planning (Suddendorf & Corballis, 2007), and likely explains why participants who were exposed to backward illusory motion experienced more past-oriented and non goal-oriented thoughts. That backward vection was more likely to trigger stimulus-related thoughts than forward vection, might also be because of the propensity for participants in this condition to be more receptive to memories being triggered even by the general experimental context. Backward illusory motion was associated with MW thoughts that stretched more distally into participants’ autobiographical past compared to the future-thinking thoughts evoked by forward illusory motion. This finding dovetails with previous research showing that thoughts about the past are more temporally distant than other MW episodes (Berntsen and Jacobsen, 2008, Stawarczyk, 2018). This is possibly because, in contrast to future-oriented thoughts, they do not primarily serve an adaptive short-term goal-planning function (Baird et al., 2011, Klinger, 2013, Stawarczyk et al., 2011).

We also observed properties of MW thoughts that did not vary as a function of the direction of vection. As predicted, participants’ MW predominantly involved thoughts of real, as opposed to fantasy, subject matter, that was self-oriented. This finding accords with previous research that found past-oriented thoughts typically consist of autobiographical memories of real events (D’Argembeau and Van der Linden, 2004, Viard et al., 2011) and that future-oriented thoughts are often mental simulations of meaningful events pivoting around one’s personal concerns (Andrews-Hanna et al., 2013, Klinger, 2013, Stawarczyk et al., 2013). We did not find the expected higher level of detail for the thoughts of participants exposed to backward vection, that were mostly past-oriented (Andrews-Hanna et al., 2013, Fox et al., 2013, Smallwood et al., 2016). In our study, the MW thoughts that occurred in both vection conditions were coherent and detailed perhaps because of the nature of our paradigm, which was unencumbered by an accompanying vigilance task, thus permitting even non-memory future thoughts to be experienced in a fully developed fashion. Moreover, recent research has reported that details generated as a function of induction and task did not vary for memories and imagination of future events (Schacter, Benoit, & Szpunar, 2017).

Overall, our findings suggest that while illusory motion evidently affected the temporal orientation of thoughts when MW, as previously found by Miles, Karpinska, et al. (2010), the effect of directional illusory motion on thought features may not be directly related to the vection stimuli, but instead to the inherent properties of the thoughts experienced.

It has been shown in behavioural and neuroimaging research that remembering past experiences and imagining future events seem to rely on a common underlying process of constructing internal representations of scenes (Hassabis and Maguire, 2007, Zeidman and Maguire, 2016). Our finding that scenes featured prominently as the main form of visual imagery used by participants accords with this view. While there is general consensus that a “core” neural network underlies both retrospective and prospective thinking, of which a brain structure called the hippocampus is a major component, research has suggested that this network might be fractionated into subcomponents interacting with additional networks that differentially activate for different thought features (Kucyi, 2018, O’Callaghan et al., 2015, Smallwood et al., 2016, Karapanagiotidis et al., 2016). Our behavioural results indicate that although MW thoughts involving past and simulated future events were comparable in terms of features such as the strategy used (visual – typically scenes) and viewpoint (first-person), they also displayed distinctive characteristics including temporal distance and goal-orientation. It is possible that these different features have distinct neural signatures and are associated with activity and patterns of connectivity in distinct subcomponents of core neural networks (Kucyi, 2018, O’Callaghan et al., 2015, Karapanagiotidis et al., 2016, McCormick et al., 2018).

Our results may also have relevance for understanding how embodied spatiotemporal information underlies covert processes such as MTT and MW (Barsalou, 2008). Dijkstra, Kaschak, and Zwaan (2007) showed that bodily movements through space influenced the rate at which autobiographical memories are recalled as well as the emotional content of the memories. Similarly, Aksentijevic et al. (2019) demonstrated that backward motion-induced MTT to the past improved memory recall for different types of information. Moreover, results from Miles, Karpinska, et al. (2010) suggested that the mere perception of backward and forward motion facilitates past and future-oriented thinking respectively. Results from our study further inform this growing field of research which suggests that MTT may be based upon representations of bodily experiences in perception and motor action, by tentatively showing that the direction of illusory motion through space modulates not only the temporal locus of spontaneous mental activity but also its phenomenological features. Nevertheless, it must be noted that results from the present experiment do not directly inform about the representational mechanisms underlying spatiotemporal integration during MW (Barsalou, 2008).

Other caveats should also be borne in mind. We mirrored some of the key parameters of Miles, Karpinska, et al.’s (2010) experiment, including testing the same number of participants in each group, employing a between-groups rather than a within-subjects design, and harvesting a limited number of thoughts from each participant. Larger sample sizes and collection of more thoughts over a longer timescale should be considered in future studies and in particular could inform about individual variability in MW (Palmisano et al., 2015, Seno et al., 2010, Seno et al., 2011). Moreover, as we did not measure participants’ subjective perceived strength of the illusory motion, it is possible that the visual stimuli we employed differentially affected participants’ thoughts. Moreover, it is natural to question whether a visual display is representative of real-life motion. Although the visual stimuli we used were previously found to induce illusory self-motion (Miles et al., 2010, Seno et al., 2012), they lacked the richness of information that typically accompanies movement in daily life. Stimuli with more proprioceptive, haptic and auditory information could be employed in order to optimally represent physical movement (Riecke, Väljamäe, & Schulte-Pelkum, 2009). As such, virtual reality could be utilised to study MW, as it has been shown to effectively induce illusory self-motion and allow participants to easily ‘get lost’ and decouple from the external environment (Riecke, Schulte-Pelkum, Avraamides, Heyde, & Bülthoff, 2006).

Finally, recent research has found that stimulus-dependent thoughts (equivalent to ‘stimulus-related’ in our study) and stimulus-independent thoughts differ in terms of their temporal orientation, with stimulus-independent thoughts being more future-oriented than stimulus-dependent thoughts (Maillet et al., 2017, Plimpton et al., 2015). This could be because external stimuli might trigger involuntary memories, thereby rendering past-oriented thoughts more ‘stimulus-related’ (Mace, 2004, Schlagman et al., 2006). The difference in the temporal orientation of stimulus-related and stimulus-independent thoughts was, however, only evident when using certain verbal cues (Maillet et al., 2017), making it difficult to generalise these findings to our data given the dissimilar nature of the stimuli. Nevertheless, future work should probe this interesting issue further.

## 5 Conclusions

Overall, our findings align with the view that temporal thinking may have a sensory-motor grounding given our finding that apparent movement in space influenced the direction and nature of participants’ self-generated thoughts. As such, we corroborated and extended the findings of Miles, Karpinska, et al. (2010), presenting new insights into the effect of illusory motion on MW using a novel experience-sampling paradigm that exposed participants to illusory motion and freed them from any cognitive load. Despite our task being clearly different from the most widespread tasks used to capture MW (e.g. Andrews-Hanna et al., 2013, Baird et al., 2011, Stawarczyk et al., 2013, Smallwood et al., 2016), the high proportion of MW thoughts experienced by participants in our study, and the simplicity of the paradigm, render it an ecologically valid approach that could be readily applied in a variety of contexts. For example, it could be used as a means to test autobiographical memory and future-thinking in patients who might not easily tolerate tasks assessing these functions directly. Similarly, paediatric participants could be tested in order to gain insights into the developmental trajectories of MW, autobiographical memory and future-thinking in a non-demanding way. More work is needed in order to optimise this paradigm and adapt it to specific cohorts. Finally, vection would also be a useful means of examining the neural substrates of MW and MTT during neuroimaging in order to investigate whether different phenomenological properties of thoughts are associated with distinct neural signatures and connectivity within subcomponents of the established brain networks. Arguably, therefore, the present paradigm represents an effective way of manipulating mind-wandering, a phenomenon that is pivotal to our cognition, and about which we have still much to learn.

## Funding

This work was supported by a Wellcome Principal Research Fellowship to E.A.M. (101759/Z/13/Z), the Centre by a Centre Award from Wellcome (203147/Z/16/Z), and by University College London’s Institute of Cognitive Neuroscience MSc in Cognitive Neuroscience Programme.

## Author statement

**Alice Liefgreen:** Conceptualization, Investigation, Data curation, Formal analysis, Writing. **Marshall A. Dalton:** Conceptualization. **Eleanor A. Maguire:** Conceptualization, Data curation, Formal analysis, Writing.

## Declaration of Competing Interest

The authors declare that they have no known competing financial interests or personal relationships that could have appeared to influence the work reported in this paper.

## Data Availability

Requests for the data can be sent to e.maguire@ucl.ac.uk.
